# Identification of stress response proteins through fusion of machine learning models and statistical paradigms

**DOI:** 10.1038/s41598-021-99083-5

**Published:** 2021-11-05

**Authors:** Ebraheem Alzahrani, Wajdi Alghamdi, Malik Zaka Ullah, Yaser Daanial Khan

**Affiliations:** 1 Department of Mathematics, Faculty of Science, King Abdulaziz University, P. O. Box 80203, Jeddah, 21589 Saudi Arabia; 2 Department of Information Technology, Faculty of Computing and Information Technology, King Abdulaziz University, P. O. Box 80221, Jeddah, 21589 Saudi Arabia; 3 Department of Computer Science, University of Management and Technology, Lahore, 54770 Pakistan

**Keywords:** Proteomics, Sequencing

## Abstract

Proteins are a vital component of cells that perform physiological functions to ensure smooth operations of bodily functions. Identification of a protein's function involves a detailed understanding of the structure of proteins. Stress proteins are essential mediators of several responses to cellular stress and are categorized based on their structural characteristics. These proteins are found to be conserved across many eukaryotic and prokaryotic linkages and demonstrate varied crucial functional activities inside a cell. The in-vivo, ex vivo, and in-vitro identification of stress proteins are a time-consuming and costly task. This study is aimed at the identification of stress protein sequences with the aid of mathematical modelling and machine learning methods to supplement the aforementioned wet lab methods. The model developed using Random Forest showed remarkable results with 91.1% accuracy while models based on neural network and support vector machine showed 87.7% and 47.0% accuracy, respectively. Based on evaluation results it was concluded that random-forest based classifier surpassed all other predictors and is suitable for use in practical applications for the identification of stress proteins. Live web server is available at http://biopred.org/stressprotiens, while the webserver code available is at https://github.com/abdullah5naveed/SRP_WebServer.git

## Introduction

Proteins are macromolecules (also known as large biomolecules) and are comprised of usually one or multiple amino acid chains. Protein is a major nutrient that is essential for tissue development. Proteins are made up of several different amino acids that are bound together in form of a polypeptide chain. There are twenty distinct building blocks widely found in plants and animals that make protein from these amino acids. The specific number, composition, and sequence of amino acids make each protein unique^1^.

First coined in 1936, the terminology “Stress” describes the relationship between a force and the resistance to fight or counter that force. Stress is a sensation of physical or emotional discomfort that can make someone feel angry, irritated, disturbed, frustrated, depressed, or nervous. The founder of stress theory, Hans Selye described stress as the nonspecific response of any demand upon a body^2^.

At the cellular level stress proteins are generated as a response to change in the activity or the state of a cell. This change typically involves various inconsistencies in movement, secretion, gene expression, or enzyme production causing a stressful condition. Stress is usually but not always an external condition such as amino acid deprivation, humidity, temperature, or ionizing radiation^3^. Stress proteins include a cohort of proteins such as protein disulfide isomerases, heat shock proteins, peptidyl-propyl isomerases and RNA chaperone proteins^4^. The association of stress proteins with many human diseases is evident in literature including cardiac diseases (such as heart attack) and major neurodegenerative diseases i.e. Alzheimer's disease, Huntington’s disease, and Parkinson’s disease^5,6^. Neurodegenerative diseases are those disorders that are known to involve gradual degeneration of the central or peripheral nervous system (CNS or PNS). Stress proteins are principal mediators of multiple responses to cellular stress and are sub-divided according to their mechanism of action into two categories^4^. One category of these stress proteins is activated only under cellular stressed conditions, whereas others are activated to enhance cell survival in both stressed and normal cellular functions^7^.

These proteins are found to be conserved across many eukaryotic and prokaryotic linkages and demonstrate varied functional activities inside a cell. For instance, mutations in DNA encoding stress proteins of Drosophila are hindered, with the mitotic division and proteasome-mediated protein degradation^8^, affecting their survival at elevated temperatures.

Classic examples of stress proteins include heat shock proteins or molecular chaperones that help to repair cellular damage^9,10^. Moreover, Chaperones can significantly alter disease progression in the case of chronic injuries, DNA damage, and age-related cellular dysfunction^3^. Their tissue specificity and selective induction exhibit their potential evolution through micro-environmental changes despite their ubiquity in all organisms. Also, to enhance cell survival, stress response proteins^11^ modulate immune responses and function in tissue and organ trauma. Clinical implications of heat shock proteins account for their structural and functional understanding and their potential roles in therapy or treatment.

Herein, a method is proposed for sequence-based identification of stress response proteins, with the help of diverse machine learning approaches to supplement the more costly wet lab methods. Classifiers are developed for the identification of stress proteins which are rigorously evaluated using well-known model accuracy metrics. The stepwise methodology involves the following steps^12^ (1)—a robust benchmark dataset collection, (2)—feature extraction, (3)—training of machine learning models, (4)—model testing and evaluation, and (5)—deployment of a model using a publicly available web server.

## Materials and methods

**Figure 1:**
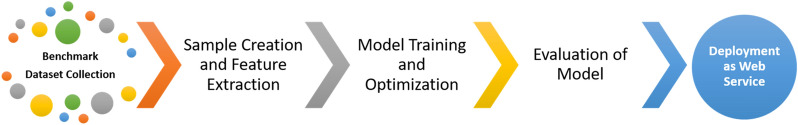
Proposed stepwise approach.

This section explains the proposed methodology based on the described stepwise approach. Each step of the described model is illustrated in Fig. 1. Firstly, a robust diverse, and homology restricted dataset is accumulated. In the next step, a comprehensive feature extraction methodology is formulated that ensures that all the crucial obscure features for deciphering the attributes of each protein have been extracted. Subsequently, based on the obtained feature vectors machine learning models are trained. Consequently, the precision of each model is calibrated to identify the most accurate model. Ultimately, the most assiduous model is integrated into a web server for public use.

### Benchmark dataset collection

**Figure 2:**
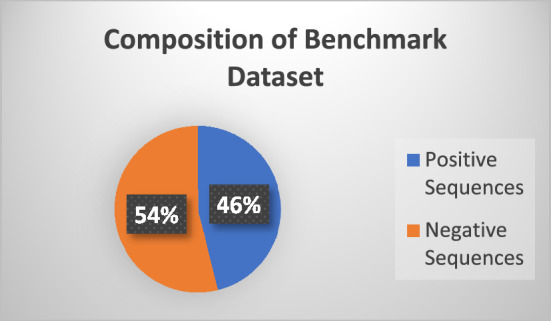
Ratio of positive AND negative dataset.

The protein sequences of the benchmark dataset were meticulously extracted from the well-known UniProt database for proteins^13^. Reviewed data of stress protein sequence of different cell lines (organisms) were retrieved from the UniProt dataset using the UniProt keyword “Stress Response” labelled as KW-0346 in the database, where the query resulted in 7092 reviewed positive protein sequences. Again, using the inverse query, 7500 reviewed negative protein sequences were retrieved. The data contained sequences of proteins in FASTA format, where each sequence was comprised of amino acids letters. Furthermore, all the sequences were of non-uniform length. However, this was the raw data, which might have been containing homologous sequences. Thus, to cater for this, redundancy from the dataset was removed using the CD-HIT suite to an acceptable level. A cutoff value was set at 0.7 to form homologous clusters with similarities greater than or equal to 70%, as reported by^14^. Ultimately, 6140 clusters were formed out of the positive samples while 7163 were formed for negative samples. A representative protein sequence was taken from each cluster. The ratio of positive and negative proteins sequence is illustrated in Fig. 2.

### Sample encoding

Machine-learning algorithms learn based on numeric representations of data, instead of raw sequences, as expounded in^15,16^]. Thus, to represent sequences as vectors, the pseudo amino acid composition (PseAAC) was proposed^17^. The idea of PseAAC is popular in bioinformatics research^18,19^ and has been used in numerous bio-medicine and medication improvement studies^20,21^ as well as other disciplines of computational proteomics. An extensive rundown of references is provided in a survey paper^22^. Since it has been generally and progressively utilized, many profound open-access projects^23,24^ were developed to create different methods of feature extractions using PseAAC. Enlivened by the achievements of utilizing PseAAC for feature extraction, multiple predictors were proposed by researchers^25,26^. Specific components for protein groups have been utilized for enabling vector encoding of samples to be processed through machine learning algorithms. These vector encoding techniques are used in various genomic research contributions as illuminated in^13^ including, a robust web server called Pse-In-One^27^. Both protein/peptide and Protein groups can be used to make a perfect fixed scale feature vector as^8^

$${\mathrm{L}}_{\upxi =7}(\mathrm{I})=[{\Psi }_{1}{\Psi }_{2}...{\Psi }_{\mathrm{u}}...{\Psi }_{\Omega }]^{\mathrm{D}}$$

The parts Ψj (j = 1, 2, ⋯, Ω) of the sequence are considered as a method of incorporating the properties of the Protein's sequence. We encoded the PseAAC values using the simplest encoding as shown in Table 1.

**Table 1:** Amino acid encodings for feature extraction.

X	A	C	D	E	F	G	H	I	K	L	M	N	O	P	Q	R	S	T	U	V	W	Y
0	1	2	3	4	5	6	7	8	9	10	11	12	13	14	15	16	17	18	19	20	21	22

For feature extraction, we used the structure of measurable statistical moments to capture the characteristics and measurements as discussed in the following sections.

### Statistical moments

Statistical moments were used in this study for feature extraction. Arbitrary statistical moments explain different aspects of the dataset defined moments defining functions and the distribution polynomial. Some specific moments were incorporated such as “Raw, Central and Hahn moments”^28,29^. The raw moment exhibits location and scale variance are used for mean calculation as well as determination of dataset asymmetry based upon probability distribution. Central moments are location invariant because centric calculations are performed. They two provide information regarding mean, variance, and distribution of data along with the mean^30,31^. Hahn moments are used to calculate the variation of size and position based on “Hahn Polynomials”. All these moments provide significant information about the sequence order and composition of data^32^. The benefit of selecting these measurable moments is the availability of the sensitive hidden patterns of peptide sequence uncovered by these moments^33^. Some of these moments especially the Hahn moments require a two-dimensional square matrix as input. For this purpose, a one-dimensional protein sequence is mapped onto a two-dimensional array. A square matrix, L′, is formed which can be expressed as

$${L}^{^{\prime}}=\left(\begin{array}{ccc}{c}_{11}& \dots & {c}_{1h}\\ \vdots & \ddots & \vdots \\ {c}_{g1}& \cdots & {c}_{gh}\end{array}\right)$$

where each ${c}_{ij}$ is an amino acid residue. The square root of the length of each sequence is computed and ceiled, and a square matrix of obtained value is formed. Each element of that square matrix is filled with residues of the respective sequence, sequentially. Moments up to the degree of 3 were computed and using the components of L′ the raw moments are determined as

$${G}_{xy}={\sum }_{l=1}^{h} {\sum }_{n=1}^{h\sum }{l}^{x}{n}^{y}{\beta }_{ln}$$

where (*l* + *n*) represents the degree of moments, while up to 3-degree moments are *G*_*00,*_* G*_*10*_*, G*_*20*_*, G*_*30*_, *G*_*01*_*, G*_*11*_*, G*_*21*_*, G*_*02*_*, G*_*12*_*,* and *G*_*03*_. Further, the central moments are determined as

$$H_{{xy}} = \sum _{{l = 1}}^{h} \sum _{{n = 1}}^{{h\sum }} (l - \bar{a})^{i} (n - w)^{y} \beta _{{ln}}$$

Furthermore, discrete Hahn moments effectively enrol for an even-dimensional information connection. Discrete Hahn moments require square cross-segment as information. The computing of Hahn moments does not change any meaning of the data, thus, due to this orthogonality, the Hahn moments are reversible and the sequence could be regenerated by using the inverse function of Hahn moments. These moments comprise sequence composition and relative positioning of amino acid residues. Hahn moments were computed through

$${N}_{h}^{z,t}(j,A)=(A+T-1{)}_{a} \times {\sum }_{i=0}^{h}(-1{)}^{i}\frac{(-h{)}_{k}(-j{)}_{i}(2A+z-t-a-1{)}_{i}}{(A+t-1{)}_{i}(A-1{)}_{i}}\frac{1}{I!}$$

where the definition of the operators used is discussed in detail by Akmal et al.^34^. The orthogonal normalized Hahn moments for 2D data are further computed as

$${E}_{xy}={\Sigma }_{n=0}^{H-1} {\Sigma }_{l=0}^{H-1} {\beta }_{xy}{e}_{x}^{\stackrel{\sim }{a,t}}\left(n,H\right){e}_{y}^{\stackrel{\sim }{a,t}}\left(l,H\right)$$

$$\text{g, h= 0,1,}\cdots H\text{-1}$$

Thus, for computing all the three types of moments, their respective equations were used. Each type of moment yielded 10 moments of order 3, thus, a total of 30 moments were computed for each sample.

#### Computing position relative incidence matrix (PRIM) by original and reverse sequence

The relative positioning of amino acid residues in a polypeptide chain plays a pivotal role in determining the biological function and physiochemical characteristics of that peptide. This pivotal model is based on the distinctive arrangement of proteins and the relative positions of the amino acids of the residue chain.

The relative positioning statistics of a total number of residues are used to assemble a 20 × 20 position relative incidence Matrix (PRIM), while the resultant matrix is used to extract features. The PRIM matrix is shown as

$${M}_{PRIM}=\left[\begin{array}{cccc}{M}_{1\to 1}& {M}_{1\to 2}\cdots & {M}_{1\to y}\cdots & {M}_{1\to 20}\\ {M}_{2\to 1}& {M}_{2\to 2}\cdots & {M}_{2\to y}\cdots & {M}_{2\to 20}\\ {M}_{x\to 1}^{\vdots }& {M}_{x\to 2}^{\vdots }\cdots & {M}_{x\to y}^{\vdots }\cdots & {M}_{i\to 20}^{\vdots }\\ {M}_{A\to 1}^{\vdots }& {M}_{A\to 2}^{\vdots }\cdots & {M}_{A\to y}^{\vdots }\cdots & {M}_{A\to 20}^{\vdots }\end{array}\right]$$

where each element ${M}_{i\to j}$ is the sum of all positions of jth amino acid, relative to the first occurrence of ith amino acid. Through this 20 × 20 matrix (as there are 20 amino acids), a total of 400 coefficients are produced by the PRIM. For reverse position relative incidence matrix (RPRIM), the same process is used on the reverse proteomic sequence and RPRIM is shown as

$${M}_{RPRIM}=\left[\begin{array}{cccc}{M}_{1\to 1}& {M}_{1\to 2}\cdots & {M}_{1\to y}\cdots & {M}_{1\to 20}\\ {M}_{2\to 1}& {M}_{2\to 2}\cdots & {M}_{2\to y}\cdots & {M}_{2\to 20}\\ {M}_{x\to 1}^{\vdots }& {M}_{x\to 2}^{\vdots }\cdots & {M}_{x\to y}^{\vdots }\cdots & {M}_{i\to 20}^{\vdots }\\ {M}_{A\to 1}^{\vdots }& {M}_{A\to 2}^{\vdots }\cdots & {M}_{A\to y}^{\vdots }\cdots & {M}_{A\to 20}^{\vdots }\end{array}\right]$$

To reduce the dimensionality of both the 20 × 20 matrix and to extracting more significant and meaningful information from PRIM and RPRIM, again 30 moments were computed for both matrices.

#### Determination of frequency vector

PRIM and RPRIM mainly provide information regarding the relative positioning of the residues in the amino acid sequences, however, amino acid frequencies in a sequence also play an important role. To elucidate the compositional confirmation of a primary sequence, another vector is formulated namely the frequency vector. The vector is of length 20, where each index i represents the *i*th amino acid from A to Y and each coefficient in this vector is used to measure the frequency occurrence of the corresponding amino acid. The frequency vector is represented as

$$\xi =\{{\tau }_{1,}{\tau }_{2,}..{.}_{,}{\tau }_{20}\}$$

where each ${\tau }_{i,}$ characterizes the frequency occurrence of that respective *i*th amino acid.

#### Computing accumulative absolute position incidence vector (AAPIV) by original and reverse sequence

The frequency vector is only used for extraction of the information of the composition of amino acids, whereas, the PRIM and RPRIM only provide information of relative amino acid positioning. To encode the absolute position of amino acids in a sequence, the Accumulative Absolute Position Incidence Vector (AAPIV), is used. It provides an estimate of the absolute positioning of residues. It computes the ordinal value of each residue and accumulates this ordinal value into a 20-length vector at the respective coefficient where each index represents the respective amino acid from A to Y. Thus, an arbitrary *i*th element of AAPIV is calculated as

$${\mu }_{i}={\sum }_{k=1}^{n}{p}_{k}$$

where ${p}_{k}$ represents the ordinal value of an arbitrary occurrence of *i*th amino acid. Similarly, Reverse Accumulative Absolute Position Incidence Vector (RAAPIV) is computed based on the same mechanism but with reversed sequence. More obscure features are unravelled by its enumeration. Generic representation of AAPIV and RAAPIV could be seen as

$$\Lambda =\{{\eta }_{1},{\eta }_{2},{\eta }_{3},...,{\eta }_{20}\}$$

### Model training and optimization

This study is focused on a specific type of protein and pertaining to the stress response. Three different classification algorithms were analyzed for the prediction of stress response proteins. A feature vector was assimilated using the raw, central, and Hahn moments of the two-dimensional depiction of protein arrangement along with PRIM moments, RPRIM moments, Frequency Vector, AAPIV, and RAAPIV. This yielded a feature vector of length 150, which was further input to all three classification algorithms.

#### Random FOREST

**Figure 3:**
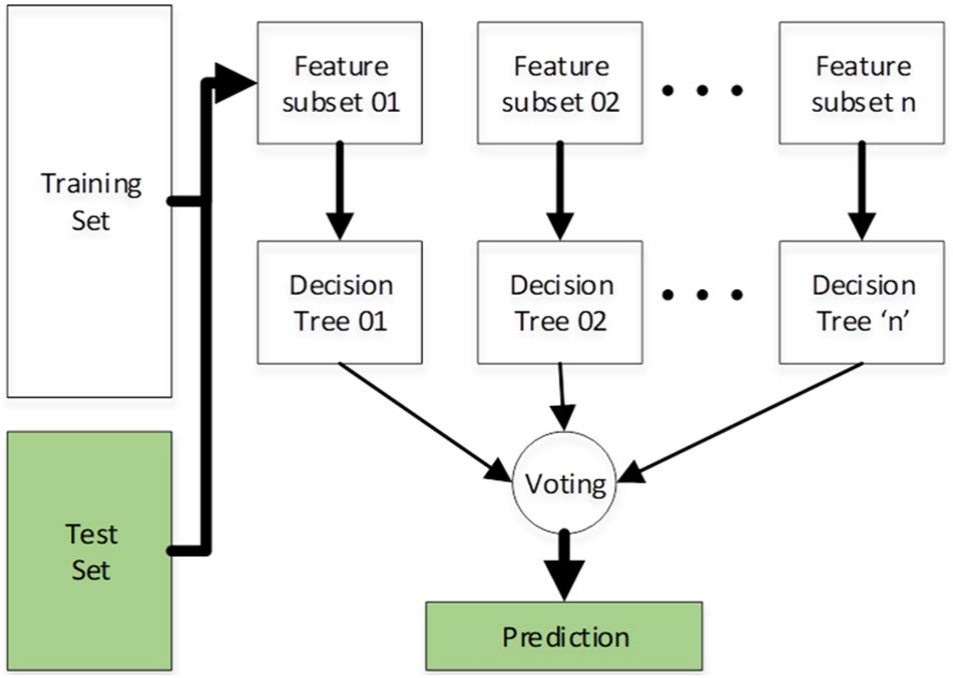
Architecture of random forest classifier for the proposed prediction model.

Firstly, the Random Forest (RF) was used which is a well-known algorithm used for regression and classification problems. While training, it is operated by generating a forest of decision trees using a feature matrix and outputs the predicted class, that is the mode of the classes predicted by all trees in that forest. Random forest is non-parametric, have higher classification accuracy, and is capable of determining the set of coefficients that are most crucial for predicting the class with maximum accuracy^35^. Feature vectors as input matrix and their corresponding class labels as expected output matrix are congregated to train the random forest predictor. The architecture of Random Forest is shown in Fig. 3.

Each tree uses a subset of the vector to classify the input where *n* is the number of decision trees. A voting algorithm finally decides the actual predicted class based on the majority votes.

In the present study, RF implementation was used from Scikit-Learn, with default parameters, while n_estimators used were 50.

#### Artificial neural network

**Figure 4:**
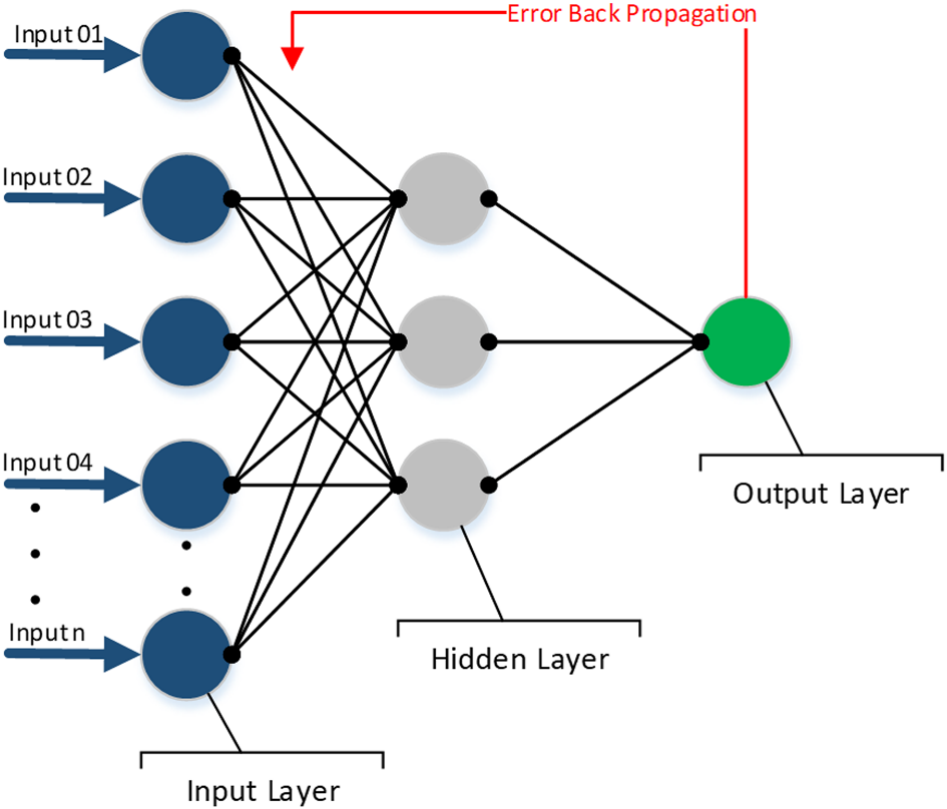
Architecture of ANN classifier for the proposed prediction model.

An artificial neural network is a connectionist network of neurons. An input neuron receives the transformed input while each subsequent neuron receives the output yielded by all the former neurons. The output of each neuron is calculated as the activated consequence of the weighted sum of the inputs to that neuron, as shown in Fig. 4. The feature vectors formed are clamped to the neural network input layer^36^. An optimized number of hidden layer neurons are used. During each epoch, the backpropagation along with gradient descent technique is used to find the most optimal neuron weights. The gradient descent method makes use of the gradients of the cost function to take a step towards the optimal solution with respect to a parameter $\theta$ as

$$\theta = \theta - a{\nabla }_{\theta }M (\theta )$$

Here, $M (\theta )$ is the objective function while $\theta \in {\mathcal{R}}^{d}, a$ is the learning rate and the gradient of the objective function is given as ${\nabla }_{\theta }M (\theta )$. The learning rate is considered to be problem-specific and its value usually relies on the cost function. It governs the step size of gradient descent at each iteration. The learning rate differential is usually a constant but it can be variable in which case it is adaptively set to find the most optimal point^37^. To find the most optimal point, the gradient is calculated for consecutive points while the weights are readjusted and the learning rate is fine-tuned^38^. When the algorithm is fully trained, it can be used to predict outcomes for unknown data.

In the present study, fully connected NN was used from Keras, with dense layers. 1 hidden layer was employed with 50 neurons, while the size of the input layer was 150 (equal to FV length). Output neurons were 2 for binary classification based on one-hot encoding. For the hidden layer, ReLU was used as an activation function while for the output layer, Sigmoid was used. The learning rate was set as 0.001.

#### Support vector machine

**Figure 5:**
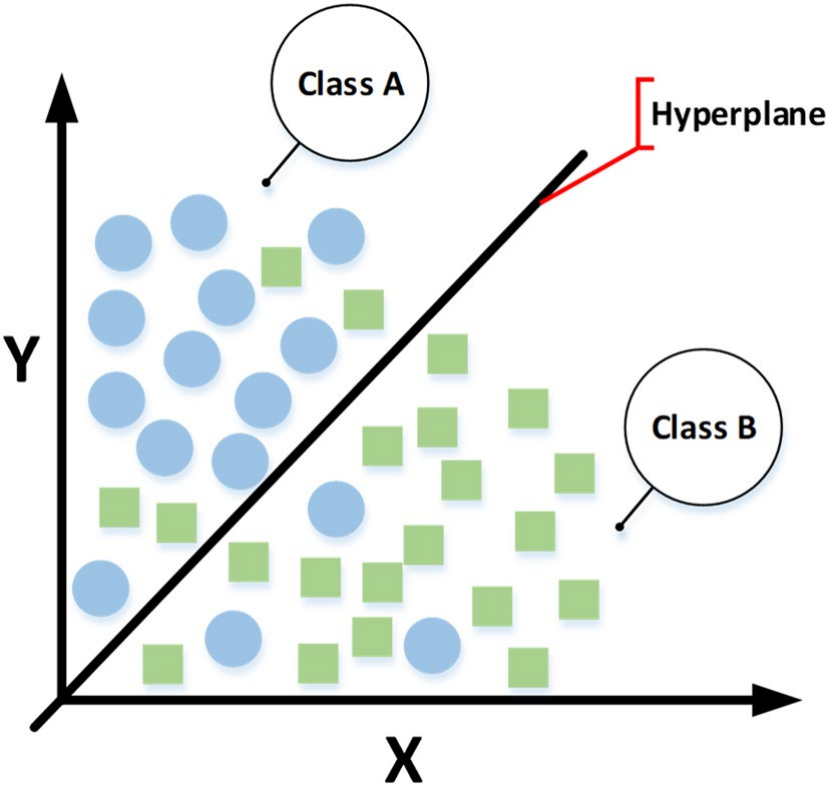
Architecture of SVM classifier for the proposed prediction model.

Lastly, a support vector machine (SVM) was used, which is also a supervised learning model, known for classification and regression tasks. SVMs has been abundantly and successfully deployed to solve numerous classification problem^39^. SVMs works on the principle of finding a hyperplane that could separate the classes of a dataset, as shown in Fig. 5.

The hyperplane is adjusted with the help of support vectors so that the distance between the hyperplane and the nearest training data points is maximal. This would provide a large margin and hence improve the accuracy by reducing the generalization error.

For the present study, Linear SVM implementation from Scikit-Learn was used, which is based on libsvm, while default parameters were employed.

## Results and discussion

Once a model is trained it is important to establish its suitability for the research community. Trained models are rigorously subjected to testing and validation using well-known validation methods. These methods help to identify the most robust, assiduous, and accurate model based on specific metrics.

### Performance estimations

The objective evaluation of a predictor is crucial to determine the performance of that predictor, however, choosing the metrics for evaluation, as well as, the method of evaluation, is a critical step. Here, in this study, four well known and well-reported metrics are used for performance evaluation which is Mathew’s Correlation coefficient (*MCC*), Specificity of the model (*S*_*p*_), Sensitivity of the model (*S*_*n*_), and finally, the Accuracy (*Acc*)^40^, as described in^14,41–48^. Here, the *MCC* is computed to reflect the stability of the model as it opts for all elements of the confusion matrix. Furthermore, the *S*_*n*_ is computed to measure the ability of the model for predicting positive samples, while *S*_*p*_ was computed for describing the ability of the model to identify negative samples^49^. In^50^, the performance estimation metrics are described as

$$\left\{\begin{array}{c}Sn= \frac{TP}{\left(TP+FN\right)}\\ Sp= \frac{TN}{\left(FP+TN\right)}\\ Acc= \frac{\left(TP+TN\right)}{TP+TN+FP+FN}\\ MCC= \frac{\left(TP\times TN\right)-\left(FP\times FN\right)}{\sqrt{\left(TP+FP\right)\times \left(TP+FN\right)\times \left(TN+FP\right)\times \left(TN+FN\right)}}\end{array}\right.$$

where $TP$ represents the number of stress response proteins that were predicted truly as stress proteins (True Positive). $FN$ represents the total number of stress response protein samples that were predicted falsely as the non-stress response protein (False negative). Also, $TN$ is the total number of non-stress response proteins that were predicted truly as non-stress (True Negatives), and $FP$ is the total number of non-stress response proteins that were falsely predicted as stress response proteins. However, these metrics are generically appropriate for binary class data. Other variants of these metrics are also proposed for multi-class data^51,52^.

After training of the proposed classifiers i.e., Random Forest Classifier, Artificial Neural Network, and SVM these predictors were thoroughly tested using rigorous validation and testing techniques i.e., self-consistency testing, jack-knife testing, independent set testing, and k-Fold Cross-Validation with k = 5 and k = 10.

#### Self-consistency test

**Figure 6:**
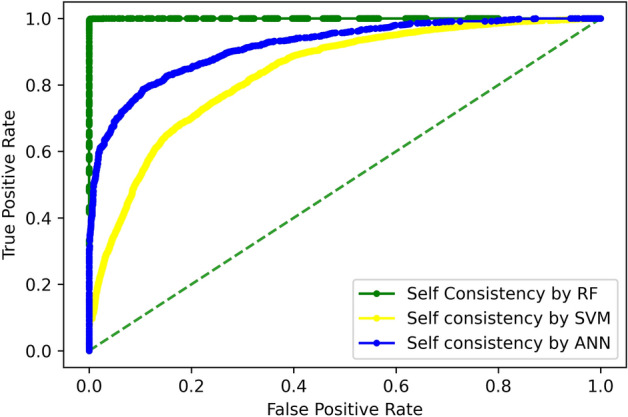
Self-consistency comparison of ROC curves.

The self-consistency test was conducted to determine the training accuracy of all classifiers using the same dataset for training and testing. The results of this test for all algorithms are shown in Table 2, which depict the overall accuracy, specificity, sensitivity, and stability of the predictive model. While the ROC curve of these predictors is Fig. 6.

**Table 2:** Self-consistency testing results of all predictors.

	TP	FN	FP	TN	Acc (%)	Sp (%)	Sn (%)	MCC
RF	6140	0	2	7161	99.9	99.9	99.9	0.99
ANN	5125	1015	708	6455	87.0	90.1	83.5	0.73
SVM	5050	1090	1534	5629	80.2	78.6	82.2	0.61

The area under the curve for the Random Forest classifier is maximal indicating that it exhibits the best performance, while the accuracy of ANN and SVM-based classifiers is 87% and 80.2%, respectively. It indicates that the random forest classifier can predict the protein class using the formulated features quite accurately as compared to the other two opted classifiers i.e., ANN and SVM.

#### jack-knife testing

**Figure 7:**
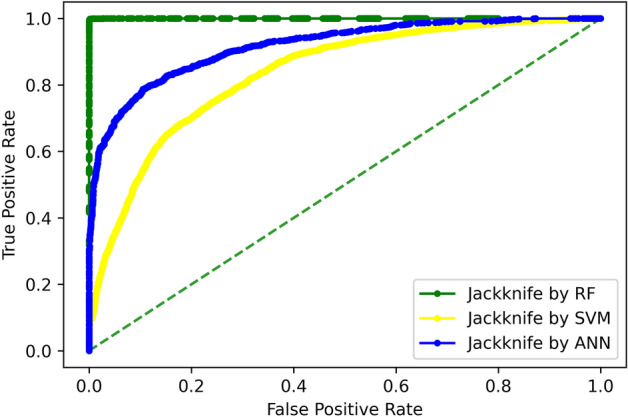
Jack-Knife comparison of ROC curves.

Jack-knife validation test bears peculiar significance as it always returns a unique output for a dataset. By using a jack-knife, intentional problems with the data such as subsampling and imbalance can be ignored. This test rigorously tests each sample as unknown data while the model is trained on the rest of the samples. At each iteration, that specific test sample is totally unseen for data, because, the model is re-initialized and re-trained. Thus, this testing is more robust than the independent dataset testing, for being testing model n-times on an un-seen sample. A jack-knife test was performed for RF, ANN, and SVM classifiers, the results are shown in Table 3. It displays the overall accuracy, specificity, sensitivity, and stability of the predictive model. The ROC curves of all three predictors as well as comparison thereof is shown in Fig. 7.

**Table 3:** Jack-knife testing results of all predictors.

	TP	FN	FP	TN	Acc (%)	Sp (%)	Sn (%)	MCC
RF	6139	1	2	7161	99.9	99.9	99.9	0.99
ANN	5125	1015	708	6455	87.0	90.1	83.5	0.73
SVM	5050	1090	1534	5629	80.3	78.6	82.2	0.61

Again, the performance of the RF classifier outperforms the rest of the classifiers.

#### Independent set testing

**Figure 8:**
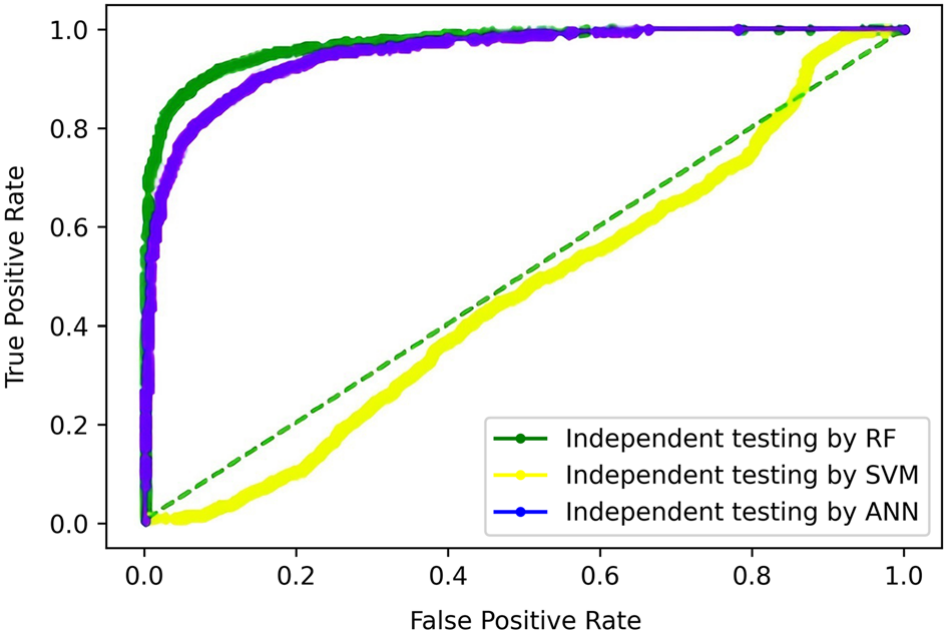
Independent testing comparison of ROC curves.

Before deploying a model for the real world, it is necessary to evaluate the performance of the model for unseen data. Independent set testing works on this principle and is performed by splitting the whole dataset into two splits. One partition is used for training, while the other is for testing. The classifiers are trained using 70% of the dataset while it's tested on the remaining 30% dataset. Table 4 displays the overall accuracy, specificity, sensitivity, and stability of the random forest, artificial neural network, and SVM classifiers. The ROC curves of all three predictors as well as comparison thereof is shown in Fig. 8.

**Table 4:** Independent dataset testing results of all models.

	TP	FN	FP	TN	Acc (%)	Sp (%)	Sn (%)	MCC
RF	1577	276	81	2057	91.1	96.2	85.1	0.82
ANN	1601	269	222	1899	87.7	89.5	85.6	0.75
SVM	1081	789	1326	795	47.0	37.5	57.8	0.048

Apparently, from the ROC curve, it is established that SVM performs unremarkably. Still, random forest exhibits the best performance while ANN shows a performance slightly inferior to RF.

#### Validation through 5-fold and tenfold cross-validation

The cross-validation test divides the dataset into k disjoint sets, where k is a predefined value chosen at the beginning of testing, and kept constant. Usually, k is kept 5 or 10 but, in this section, k is set to 5 and 10 to achieve fivefold and tenfold cross-validation. At each iteration, that specific test set is totally unseen for data, as at each iteration, the model is re-initialized and re-trained. Thus, this testing is more robust than the independent dataset testing, for being testing model k-times on un-seen data. This test helps to determine the possibility of encumbrances like underfitting and overfitting.

For fivefold, the test is iterated 5 times, in each iteration, one set (kth set) is treated as a testing set while the rest of the four sets are accumulatively used as the training set. The overall accuracy of the model is computed by taking an average of scores achieved in each iteration which is reported as the result of the cross-validation test.

**Figure 9:**
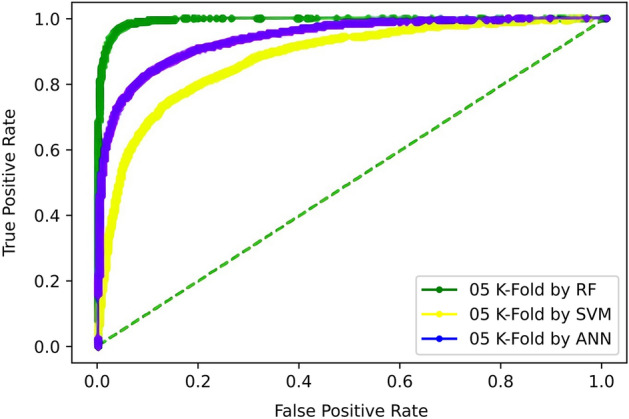
Fivefold cross-validation comparison of ROC.

The 5-Fold Cross-Validation test reveals 95.9% overall accuracy using a random-forest classifier, 87% accuracy is brought by ANN, and 79.4% accuracy is obtained by SVM as shown in Table 5. The ROC also depicts the same in Fig. 9.

**Table 5:** Fivefold cross validation result of proposed models.

	TP	FN	FP	TN	Acc (%)	Sp (%)	Sn (%)	MCC
RF	5810	330	211	6952	95.9	97.1	94.6	0.91
ANN	5125	1015	708	6455	87.0	90.1	83.5	0.73
SVM	5058	1082	1665	5498	79.4	76.8	82.4	0.58

**Figure 10:**
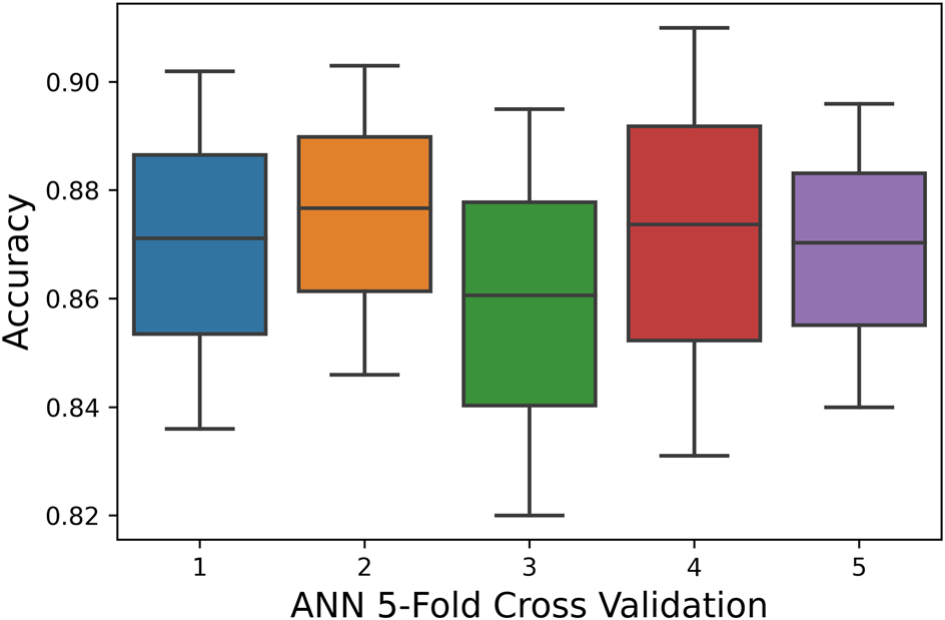
ANN fivefold cross-validation.

**Figure 11:**
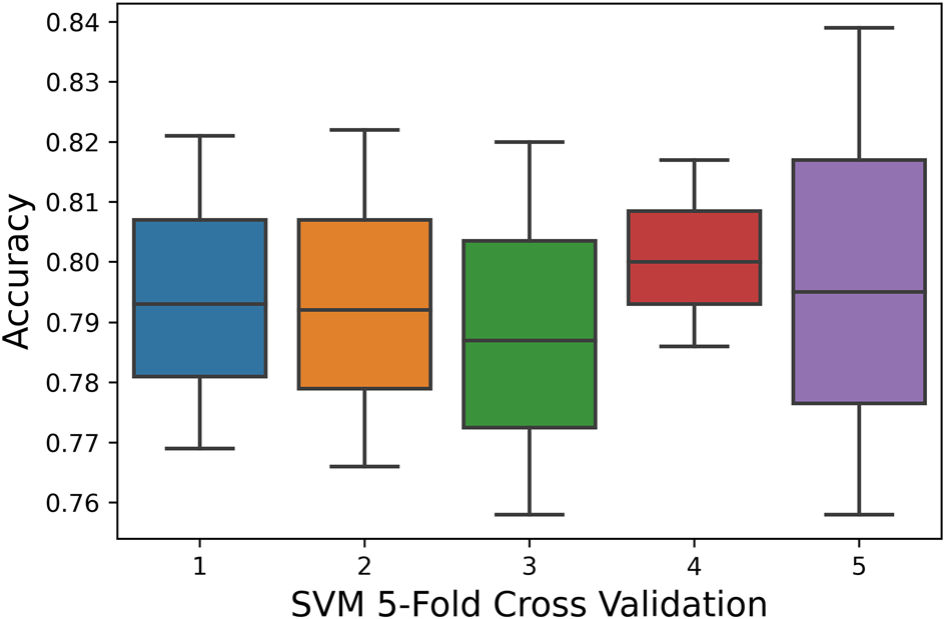
SVM fivefold cross-validation.

**Figure 12:**
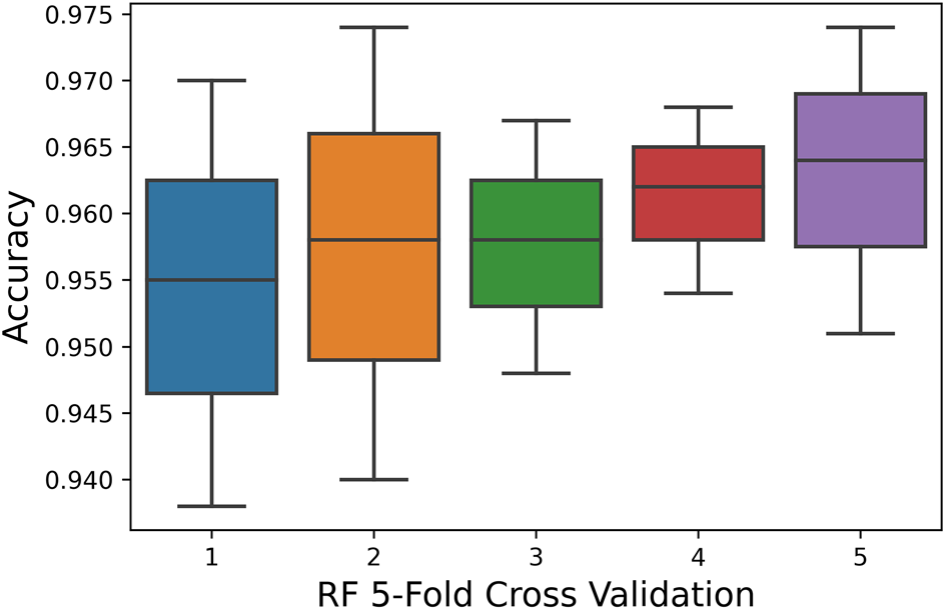
RF fivefold cross-validation.

Furthermore, the boxplot comparison of 5-Fold cross-validation of ANN, SVM and, RF is shown in Figs. 10, 11 and 12.

The boxplot shows that the peak accuracy of the RF classifier reaches 0.975 while the peak accuracies of NN and SVM are 0.91 and 0.84, respectively. Subsequently, the minimal accuracy exhibited by RF is 0.94 whereas the minimal accuracy of NN and SVM are 0.82 and 0.76, respectively. This clearly shows that RF performance is far superior to SVM and also performs NN. Random Forest classifier exhibits a stable accuracy rate over numerous partitions with a mean accuracy rate of 0.959.

**Figure 13:**
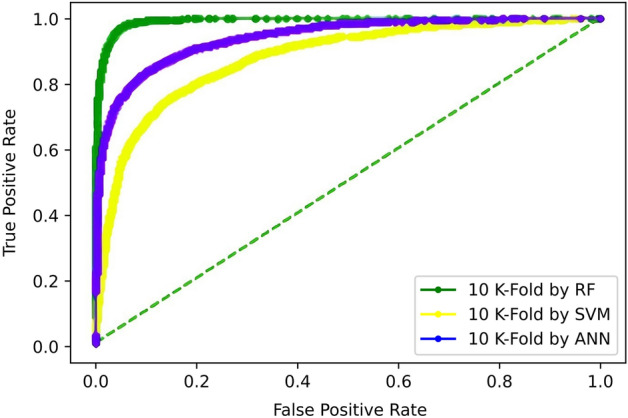
Tenfold cross-validation comparison of ROC.

For tenfold, the benchmark dataset is divided into 10 folds using k = 10, while at each iteration, 9 folds are selected for training the model while the remaining onefold is used for testing. Hence, all the partitions of the dataset are ultimately used for both testing and training. The result obtained is the average of all the scores yielded in each iteration. The results yielded show that the highest accuracy of 95.8% is exhibited by the random forest classifier. Table 6 shows the performance of all the classifiers which is also reflected by the ROC curve in Fig. 13.

**Table 6:** Tenfold cross-validation result of proposed models.

	TP	FN	FP	TN	Acc (%)	Sp (%)	Sn (%)	MCC
RF	5804	336	221	6942	95.8	96.9	94.5	0.91
ANN	5125	1015	708	6455	87.0	90.1	83.5	0.73
SVM	5054	1086	1675	5488	79.2	76.6	82.3	0.58

### Discussion

This study proposed a method based on three classifiers using Random Forest, Support Vector Machine, and Artificial Neural Network. The stress response proteins benchmark dataset was encoded with statistical features to enable better representation of the benchmark dataset which is why the dataset was clean from redundancy and the applied classifier was able to use these representations for developing better predictor models for stress response proteins. The highest performance, among the proposed three classifiers, was observed for the random forest, as shown in Tables 2, 3, 4, 5 and 6. The significance of the Random Forest classifier was ascertained by the achievement of best accuracy metrics as compared to other classifiers. As opposed to ANN and SVM, Random Forest gave the most assiduous outcomes.

For each model, five distinct tests were performed and a variance in the performance of each classification model is apparent from the results of this test. Further, the suitability of the proposed classification model is discussed based on its performance. The independent dataset testing, fivefold, and tenfold validation were performed only with the goal of performing a robust and exhaustive validation of the model, while jackknife testing helped to perform such validation which enhances the transparency of the method. The score generated by jackknife testing is always the same, whenever the method is reproduced, as this testing covers all samples of data for testing, individually. For independent dataset testing, testing was performed using 30% of data, while in fivefold and tenfold, testing was performed using random folds 5 and 10 times, respectively. The accuracies reported for k-fold are the average of k-experiments. Thus, in the case of SVM, the accuracy of independent dataset testing is quite low as compared to fivefold and tenfold. One of the main reasons could be a random sampling of the fold chosen for independent dataset testing of SVM. However, overall, it was observed that the random forest algorithm gave the most accurate and efficient results in the prediction of stress response proteins. Artificial Neural Network was observed to be passable but inferior as compared to the random forest while Support Vector Machine algorithm showed the poorest performance among all three classifiers.

The performance of RF was observed consistently to be best while SVM was consistently observed poor. Usually, the performance of different classifiers varies from data to data and one cannot compare it specifically that one algorithm is good and the other is bad. However, in the case of the present study, it could be inferred that RF is basically the combination of multiple individual decision trees to act as an ensemble, employing multiple learners to solve the same problem, whereas, the SVM classifies binary data based on a hyperplane^53–57^. Thus, the ensemble of multiple learners performed well in our case of classifying stress response proteins.

The results depict that the proposed stress response protein predictor can turn into a convenient high throughput apparatus for researchers exploring stress proteins. It may serve as the primary predictor to foresee protein stress reaction (Supplementary Information 1).

### Webserver

**Figure 14:**
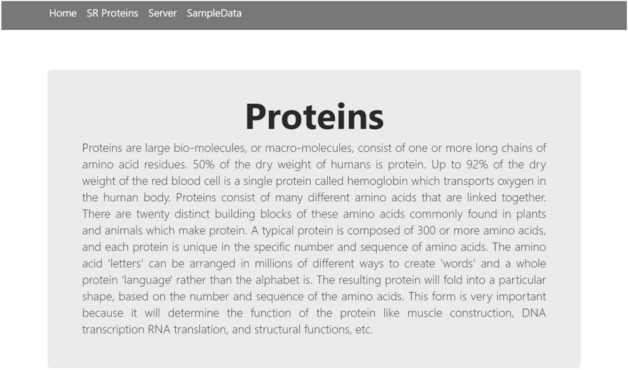
Home page of web-server.

**Figure 15:**
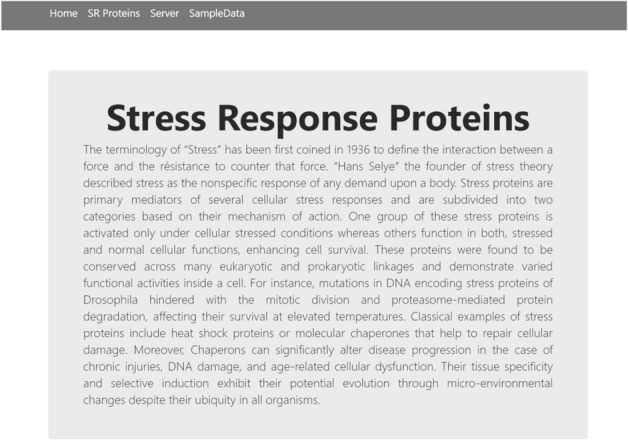
Introduction page of web-server.

**Figure 16:**
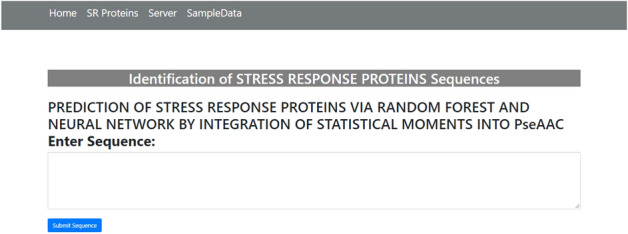
Prediction server page.

**Figure 17:**
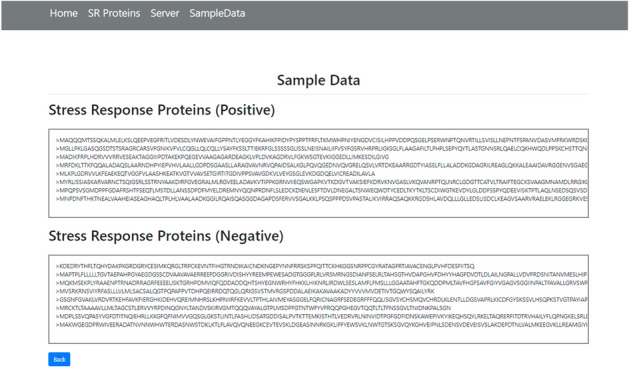
Sample data page.

**Figure 18:**
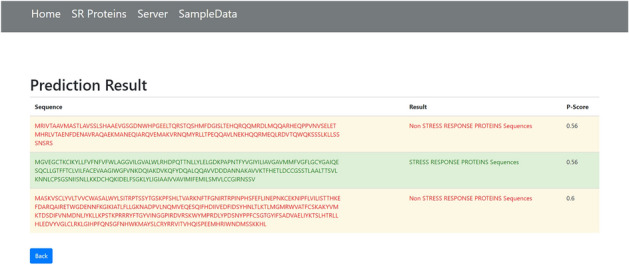
Results page of web-server.

The final phase is the implementation of the webserver. As discussed in^58,59^, the webserver enables the research community to use the latest advances. The openly available webservers show the practical usage which must be accurate and useful for prediction. For the development of a web server, the python Flask 1.0.2 framework was used to deploy models for the Stress response protein prediction. The models were implemented using Scikit-Learn, wtform 2.2.1, NumPy 1.16.3, TensorFlow 2.0.0, and Keras 2.2.4 libraries, and these libraries are used for the backend of a Webserver. Figure 14 shows the Homepage, Fig. 15 shows the introduction page, Fig. 16 shows the Prediction Server page, Fig. 17 shows the sample sequence data page, and Fig. 18 shows the results page. A live webserver is available at http://biopred.org/stressprotiens.

## Conclusion

In this study, Stress Response Protein prediction models based on RF, ANN and SVM are proposed. We used statistical measures to extract features from the benchmark dataset. Out of the three proposed models, the random forest classifier delivered the most elevated outcome. There are two aspects to the diligent outcome. Firstly, the experimental results strengthen the notion that the feature extraction model is forthcoming and yielding. Obscure patterns encompassed within the setup of amino acid residues within the polypeptide chain are uncovered by the proposed feature extraction technique. Secondly, out of all the classifiers discussed the most suitable classifier for deciphering each class based on the proposed feature set in random forest. Though ANN and SVM stimulate responses largely better than pure luck it is observed that random forest somehow exhibits the best capability for deciphering among classes. Rigorous testing techniques like cross-validation and jackknife testing ensure that the results are realistic and not an outcome of overfitting. Finally, the best performing model i.e., random forest is used to deploy as a web service to make it available for public use.

## Supplementary Information

Supplementary Information 1.

Supplementary Information 2.

## References

[1] Lesk AM (2001). Introduction to Protein Architecture: The Structural Biology of Proteins.

[2] Tan SY, Yip A (2018). Hans Selye (1907–1982): Founder of the stress theory. Singap. Med. J..

[3] Welch WJ (1992). Mammalian stress response: Cell physiology, structure/function of stress proteins, and implications for medicine and disease. Physiol. Rev..

[4] Feder ME, Hofmann GE (1999). Heat-shock proteins, molecular chaperones, and the stress response: Evolutionary and ecological physiology. Annu. Rev. Physiol..

[5] Chen X, Guo C, Kong J (2012). Oxidative stress in neurodegenerative diseases. Neural Regen. Res..

[6] Xiao X, Benjamin IJ (1999). Stress-response proteins in cardiovascular disease. Am. J. Hum. Genet..

[7] Little TJ, Nelson L, Hupp TJPO (2007). Adaptive evolution of a stress response protein. PLoS One.

[8] Rokde, C. N. & Kshirsagar, M. Bioinformatics: Protein structure prediction. In *2013 Fourth International Conference on Computing, Communications and Networking Technologies (ICCCNT). 2013. IEEE*.

[9] Chou KC, Zhang CT (1995). Prediction of protein structural classes. Crit. Rev. Biochem. Mol. Biol..

[10] Cheng J, Tegge AN, Baldi P (2008). Machine learning methods for protein structure prediction. IEEE Rev. Biomed. Eng..

[11] Hemm MR (2010). Small stress response proteins in *Escherichia coli*: Proteins missed by classical proteomic studies. J. Bacteriol..

[12] Chou K-C (2011). Some remarks on protein attribute prediction and pseudo amino acid composition. J. Theoret. Biol..

[13] Chou K-C, Shen H-B (2007). MemType-2L: A web server for predicting membrane proteins and their types by incorporating evolution information through Pse-PSSM. Biochem. Biophys. Res. Commun..

[14] Naseer S (2020). IPhosS (Deep)-PseAAC: Identify phosphoserine sites in proteins using deep learning on general pseudo amino acid compositions via modified 5-Steps rule. IEEE/ACM Trans. Comput. Biol. Bioinform..

[15] Hussain W (2020). A sequence-based predictor of Zika virus proteins developed by integration of PseAAC and statistical moments. Combin. Chem. High Throughput Screen..

[16] Naseer, S., *et al*. iPhosS (Deep)-PseAAC: Identify phosphoserine sites in proteins using deep learning on general pseudo amino acid compositions via modified 5-steps rule. 2020. 10.1109/TCBB.2020.3040747 33242308

[17] Khan SA (2019). N-MyristoylG-PseAAC: Sequence-based prediction of N-myristoyl glycine sites in proteins by integration of PseAAC and statistical moments. Lett. Organ. Chem..

[18] Ilyas S (2019). iMethylK-PseAAC: Improving accuracy of lysine methylation sites identification by incorporating statistical moments and position relative features into general PseAAC via Chou’s 5-steps rule. Curr. Genom..

[19] Barukab O (2019). iSulfoTyr-PseAAC: Identify tyrosine sulfation sites by incorporating statistical moments via Chou’s 5-steps rule and pseudo components. Curr. Genom..

[20] Malebary SJ, Rehman MS, Khan YD (2019). iCrotoK-PseAAC: Identify lysine crotonylation sites by blending position relative statistical features according to the Chou’s 5-step rule. PLoS One.

[21] Khan YD, Ahmad F, Khan SA (2013). A survey on use of neuro-cognitive and probabilistic paradigms in pattern recognition. Res. J. Recent Sci..

[22] Naseer S (2020). Sequence-based identification of arginine amidation sites in proteins using deep representations of proteins and PseAAC. Curr. Bioinform..

[23] Khan YD (2020). Sequence-based identification of allergen proteins developed by integration of PseAAC and statistical moments via 5-step rule. Curr. Bioinform..

[24] Naseer S (2021). NPalmitoylDeep-PseAAC: A predictor of N-palmitoylation sites in proteins using deep representations of proteins and PseAAC via modified 5-steps rule. Curr. Bioinform..

[25] Butt AH, Khan YD (2019). Therapeutics, prediction of S-sulfenylation sites using statistical moments based features via Chou’S 5-Step rule. Int. J. Peptide Res. Ther..

[26] Liu B (2016). repRNA: A web server for generating various feature vectors of RNA sequences. Mol. Genet. Genom..

[27] Chen W (2016). Using deformation energy to analyze nucleosome positioning in genomes. Genomics.

[28] Khan YD, Ahmad F, Anwar MW (2012). A neuro-cognitive approach for iris recognition using back propagation. World Appl. Sci. J..

[29] Khan YD (2014). Situation recognition using image moments and recurrent neural networks. Neural Comput. Appl..

[30] Butt AH (2016). A prediction model for membrane proteins using moments based features. BioMed Res. Int..

[31] Butt AH, Rasool N, Khan YD (2017). A treatise to computational approaches towards prediction of membrane protein and its subtypes. J. Membr. Biol..

[32] Khan, Y. D., *et al.* Iris recognition using image moments and k-means algorithm. 2014. 2014. 10.1155/2014/723595 PMC3995185 24977221

[33] Khan YD (2014). An efficient algorithm for recognition of human actions. Sci. World J..

[34] Akmal MA, Rasool N, Khan YD (2017). Prediction of N-linked glycosylation sites using position relative features and statistical moments. PLoS One.

[35] Hussain W, Rasool N, Khan YD (2020). Insights into machine learning-based approaches for virtual screening in drug discovery: Existing strategies and streamlining through FP-CADD. Curr. Drug Discov. Technol..

[36] Mahmood MK (2020). iHyd-LysSite (EPSV): Identifying hydroxylysine sites in protein using statistical formulation by extracting enhanced position and sequence variant feature technique. Curr. Genom..

[37] Cheng X (2017). iATC-mISF: A multi-label classifier for predicting the classes of anatomical therapeutic chemicals. Bioinformatics.

[38] Naseer S (2021). Optimization of serine phosphorylation prediction in proteins by comparing human engineered features and deep representations. Anal. Biochem..

[39] Butt AH, Khan YD (2019). CanLect-Pred: A cancer therapeutics tool for prediction of target cancerlectins using experiential annotated proteomic sequences. IEEE Access.

[40] Malebary SJ, Khan YD (2021). CONTINUA, identification of antimicrobial peptides using Chou's 5 step rule. Comput. Mater. Contin..

[41] Malebary SJ, Khan YD (2021). Evaluating machine learning methodologies for identification of cancer driver genes. Sci. Rep..

[42] Awais M (2021). iTSP-PseAAC: Identifying tumor suppressor proteins by using fully connected neural network and PseAAC. Curr. Bioinform..

[43] Awais M (2019). iPhosH-PseAAC: Identify phosphohistidine sites in proteins by blending statistical moments and position relative features according to the Chou's 5-step rule and general pseudo amino acid composition. IEEE/ACM Trans. Comput. Boil. Bioinform..

[44] Hussain W (2019). SPalmitoylC-PseAAC: A sequence-based model developed via Chou's 5-steps rule and general PseAAC for identifying S-palmitoylation sites in proteins. Anal. Biochem..

[45] Hussain W (2019). SPrenylC-PseAAC: A sequence-based model developed via Chou's 5-steps rule and general PseAAC for identifying S-prenylation sites in proteins. J. Theor. Biol..

[46] Khan YD (2019). iProtease-PseAAC (2L): A two-layer predictor for identifying proteases and their types using Chou's 5-step-rule and general PseAAC. Anal. Biochem..

[47] Khan YD (2018). iPhosT-PseAAC: Identify phosphothreonine sites by incorporating sequence statistical moments into PseAAC. Anal. Biochem..

[48] Khan YD (2018). iPhosY-PseAAC: Identify phosphotyrosine sites by incorporating sequence statistical moments into PseAAC. Mol. Biol. Rep..

[49] Malebary SJ, Khan R, Khan YD (2021). ProtoPred: Advancing oncological research through identification of proto-oncogene proteins. IEEE Access.

[50] Akmal, M. A., *et al.* Using Chou's 5-steps rule to predict O-linked serine glycosylation sites by blending position relative features and statistical moment. 2020. 10.1109/TCBB.2020.2968441 31985438

[51] Jia J (2015). iPPI-Esml: An ensemble classifier for identifying the interactions of proteins by incorporating their physicochemical properties and wavelet transforms into PseAAC. J. Theoret. Biol..

[52] Qiu WR (2017). iPhos-PseEvo: identifying human phosphorylated proteins by incorporating evolutionary information into general PseAAC via grey system theory. Mol. Inf..

[53] Kremic E, Subasi A (2016). Performance of random forest and SVM in face recognition. Int. Arab J. Inf. Technol..

[54] Huo, J., Shi, T. & Chang, J. Comparison of random forest and SVM for electrical short-term load forecast with different data sources. In *2016 7th IEEE International Conference on Software Engineering and Service Science (ICSESS)*. 2016. IEEE.

[55] Murugan A, Nair SAH, Kumar KS (2019). Detection of skin cancer using SVM, random forest and kNN classifiers. J. Med. Syst..

[56] Liao Z, Ju Y, Zou Q (2016). Prediction of G protein-coupled receptors with SVM-prot features and random forest. Scientifica.

[57] Statnikov A, Wang L, Aliferis CF (2008). A comprehensive comparison of random forests and support vector machines for microarray-based cancer classification. BMC Bioinform..

[58] Qiu W-R (2018). iKcr-PseEns: Identify lysine crotonylation sites in histone proteins with pseudo components and ensemble classifier. Genomics.

[59] Cheng X, Xiao X, Chou K-CJG (2018). pLoc-mEuk: Predict subcellular localization of multi-label eukaryotic proteins by extracting the key GO information into general PseAAC. Genomics.

